# Supplementary material for: Coastal flooding will disproportionately impact people on river deltas
Source: Nat Commun. 2020 Sep 29;11:4741. doi: 10.1038/s41467-020-18531-4 (PMC7525510; doi:10.1038/s41467-020-18531-4)
Supplement: Supplementary file 1 — Supplementary Information [file 41467_2020_18531_MOESM1_ESM.pdf]

Supplementary Information for “Coastal flooding will disproportionately impact people on river deltas” by Edmonds et al.

Supplementary Table 1: Global delta dataset sorted by various parameters. All sorting is by largest and show the top ten for each. Population density only shows the seven deltas with more than 150,000 people and greater than 10,000 people/km^2. For population density we only sort through those deltas with more than 150,000 people to avoid including densely populated that do not have many people living on them.

Sorted by Population 2017

ID	River Name	Country	Geomorphic Area (km2)	Habitable Area (km2)	Population year 2000	Population year 2010	Population year 2017	Population density year 2017 (ppl/km^2)	Population in 100-year floodplain year 2017
4027	Ganges	Bangladesh	80174.17	68849.46	77233952	103549568	105461968	1532	2329660
0001	Nile	Egypt	28344.80	26359.72	32425994	38046124	45221260	1716	3120992
1537	Yangtze	China	16993.03	13321.07	21098204	23900496	31375546	2355	1635680
4158	Mekong	Vietnam	39465.66	37595.35	15867872	17418370	17924756	477	1962159
0124	Niger	Nigeria	34553.93	32517.17	9617618	11071605	12662285	389	39278
1449	Pearl	China	5613.34	5150.60	5754290	9504483	12065796	2343	4505003
4204	Red	Vietnam	8254.05	7394.86	9892108	10212460	10623076	1437	4273955
1538	Guanhe	China	19735.02	19216.04	11551084	11751276	10541032	549	8531302
4050	Irawaddy	Myanmar	28671.67	25629.22	7281147	9841221	9715010	379	13378
4137	Chao Pharya	Thailand	4090.40	3941.90	5041581	6063585	8061067	2045	11784

Sorted by Population density 2017

2671	Neva	Russia	41.89	31.16	233369	370745	531676	17062	0
0083	St. Paul	Liberia	20.40	11.18	8212	92230	184532	16506	6
1772	Arakawa	Japan	52.26	49.59	850973	693158	753413	15194	0
1771	Edo	Japan	20.22	17.89	143140	182428	220347	12319	135
1841	Yodo	Japan	74.77	57.88	839601	677576	690775	11935	0
1514	Minjiang	China	85.50	67.16	258610	353819	684109	10187	0
1855	Ota	Japan	34.71	29.39	383684	323803	294351	10016	0

Sorted by population in 100 year floodplain

1538	Guanhe	China	19735.02	19216.04	11551084	11751276	10541032	549	8531302
1449	Pearl	China	5613.34	5150.60	5754290	9504483	12065796	2343	4505003
4204	Red	Vietnam	8254.05	7394.86	9892108	10212460	10623076	1437	4273955
0001	Nile	Egypt	28344.80	26359.72	32425994	38046124	45221260	1716	3120992
4027	Ganges	Bangladesh	80174.17	68849.46	77233952	103549568	105461968	1532	2329660
4158	Mekong	Vietnam	39465.66	37595.35	15867872	17418370	17924756	477	1962159
2700	Rhine	Netherlands	3341.13	2740.86	1950636	2098707	2075289	757	1869518
1537	Yangtze	China	16993.03	13321.07	21098204	23900496	31375546	2355	1635680
1450	Dong	China	725.47	639.34	836432	2609015	3229268	5051	1003818
1991	Agano	Japan	338.55	324.48	639318	585962	577002	1778	250278

Sorted by geomorphic area

3672	Amazon	Brazil	84429.42	58747.72	375797	646335	746287	13	5208
4027	Ganges	Bangladesh	80174.17	68849.46	77233952	103549568	105461968	1532	2329660

3419	Mississippi	USA	51124.89	39829.07	2851512	2887614	3065114	77	27438
4158	Mekong	Vietnam	39465.66	37595.35	15867872	17418370	17924756	477	1962159
0124	Niger	Nigeria	34553.93	32517.17	9617618	11071605	12662285	389	39278
0001	Nile	Egypt	28671.67	25629.22	7281147	9841221	9715010	379	13378
4050	Irawaddy	Myanmar	28344.80	26359.72	32425994	38046124	45221260	1716	3120992
3672	Amazon	Brazil	22522.62	20698.41	69834	105932	160743	8	47
3691	Orinoco	Venezuela	21059.68	14590.52	55	130	187	0	0
1538	Guanhe	China	19735.02	19216.04	11551084	11751276	10541032	549	8531302

Supplementary Table 2: Comparison between geomorphic area measurements (this study) and Syvitski and Saito. Revised area shows deltaic area from this study that only includes the active channel network. Syvitski and Saito data come from reference 42 in main article.

River	ID	Geomorphic Area (km^2)	Revised Area (km^2)	Syvitski and Saito area (km^2)	% difference with geomorphic area
Nile	1	28344		24512	15.63
Niger	124	34533	18910	17135	101.53
Colorado (California)	1122	394		634	37.85
Pearl	1449	5613		5200	7.94
Yangtze	1537	16993		35000	51.45
Huanghe	1560	6084		5710	6.55
Kolyma	2357	4108		6400	35.81
Indigirka	2363	7115		4800	48.23
Yana	2372	4188		1200	249.00
Lena	2380	21059		24000	12.25
Pechora	2629	1869		3000	37.70
Vistula	2684	1234		500	146.80
Ebro	2867	229		338	32.25
Rhone	2876	1382		1540	10.26
Po	2952	655		1050	37.62
Danube	3017	3700		4200	11.90
MacKenzie	3148	12363		13000	4.90
Yukon	3228	18295	5620	5200	251.83
Mississippi	3419	51124		38568	32.56
Brazos	3428	756	77	60	1160.00
Colorado	3431	83		38	118.42
Parana	3593	3,850		3617	6.44
Orinoco	3691	22636		35642	36.49
Amazon	3696	85667		467000	81.66
Magdalena	3713	1969		7500	73.75
Tigris–Euphrates	3805	2027		3850	47.35
Indus	3842	12763		6780	88.24
Krishna	4010	1458		2100	30.57
Godavari	4011	4791		4400	8.89
Mahanadi	4022	6608		5900	12.00
Ganges/Brahma	4027	80174		105641	24.11
Irrawaddy	4050	28671		30570	6.21
Chao Pharya	4137	4090		5500	25.64
Mekong	4158	39465		49000	19.46
Red River	4204	8254		11400	27.60
Fly	4981	3402		2800	21.50

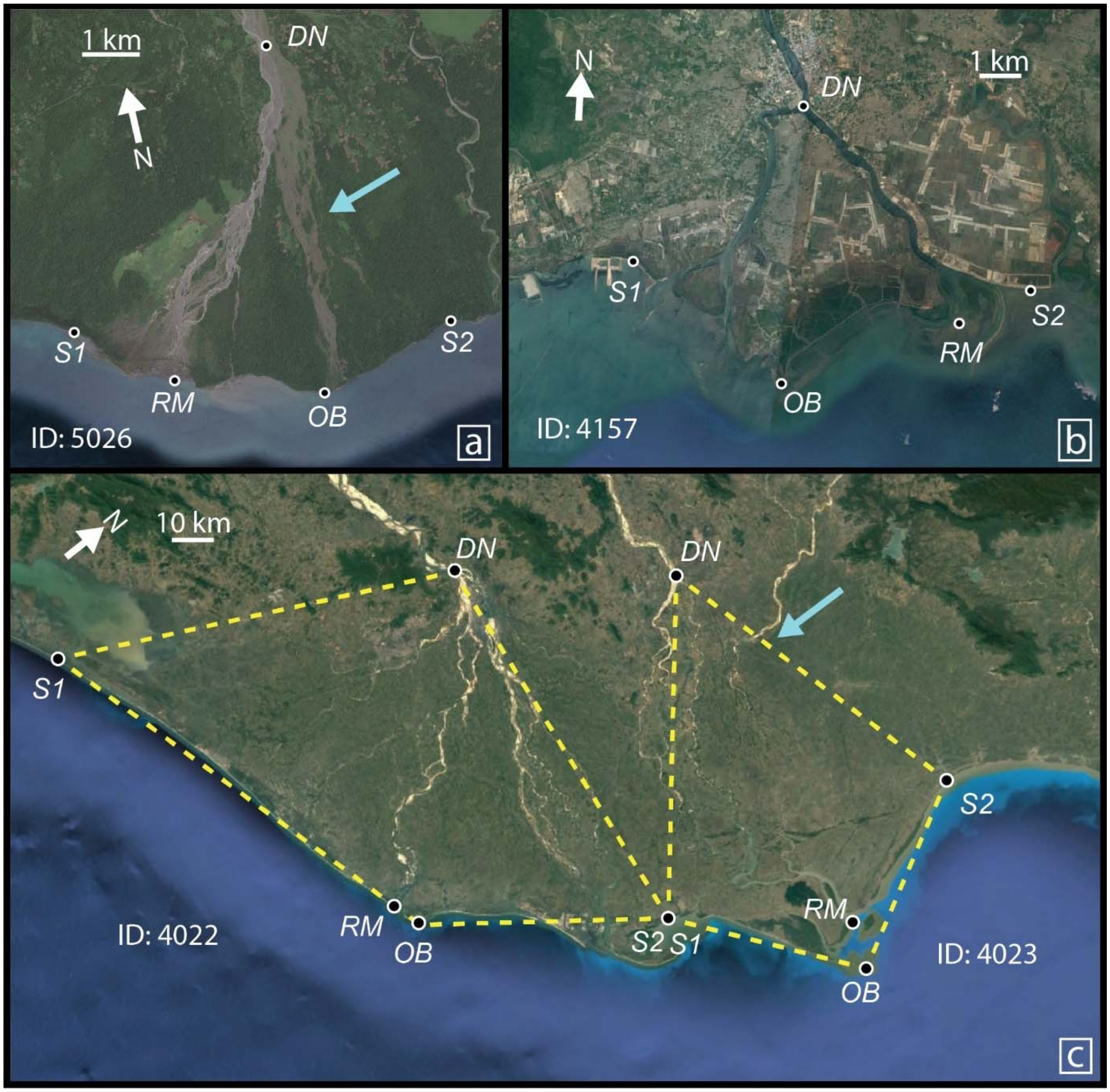

Supplementary Figure 1: Examples of the five points that define the delta polygon. (a) Example where *DN* location is chosen using relict channel (marked with blue arrow). (b) Example of human influenced delta. (c) Example of two deltas with separate IDs that share a lateral shoreline point. Delta on the right shows an example where two rivers, the one marked *DN* and one with the blue arrow, combine to form a single delta (ID: 4023). Dashed yellow lines show the delta polygons used in this study

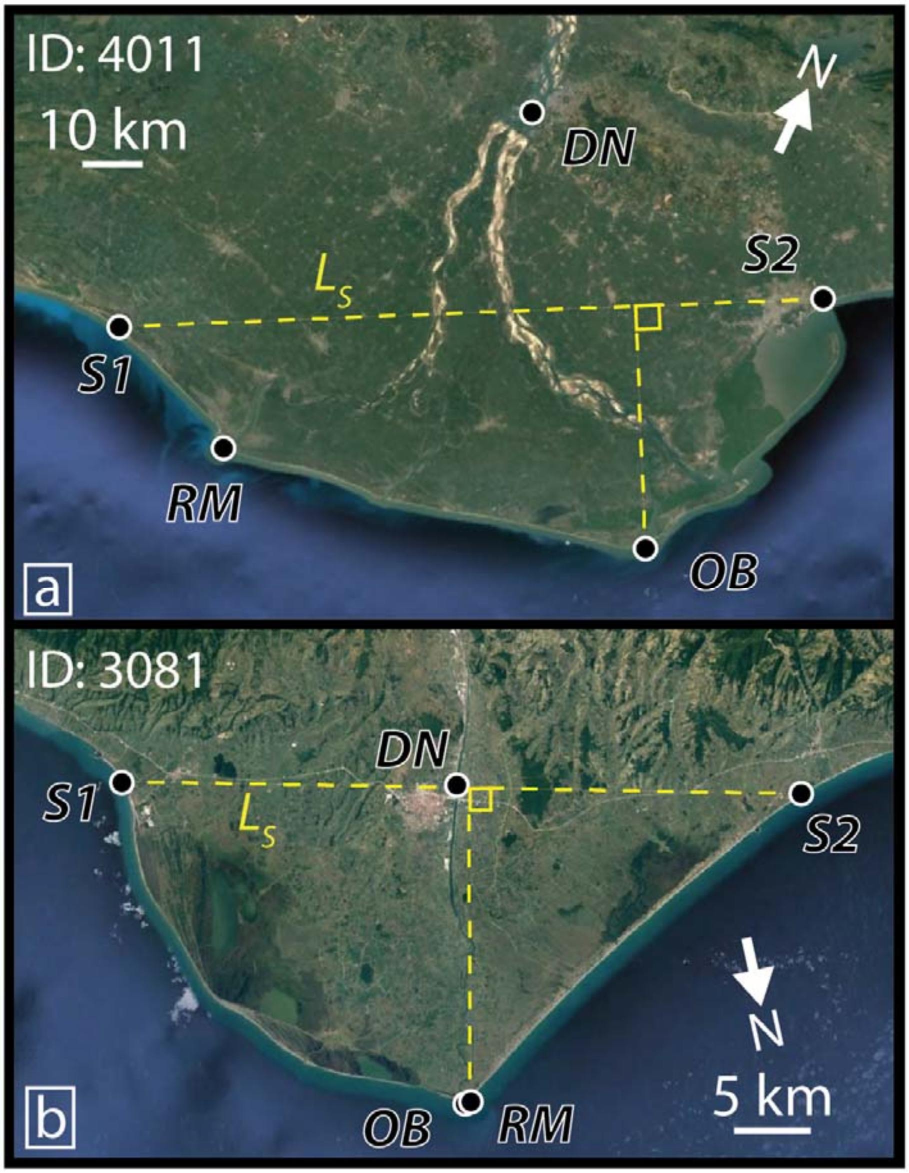

Supplementary Figure 2: Examples showing how *DN* and *OB* are determined. A local shoreline vector (L_s) is determined between points *S1* and *S2*. The delta node (*DN*) is given as either (a) the upstream most bifurcation of the parent channel the most upstream point or (b) the intersection of the main channel and L_s. If both criteria are present, then the point that is farthest upstream is selected.

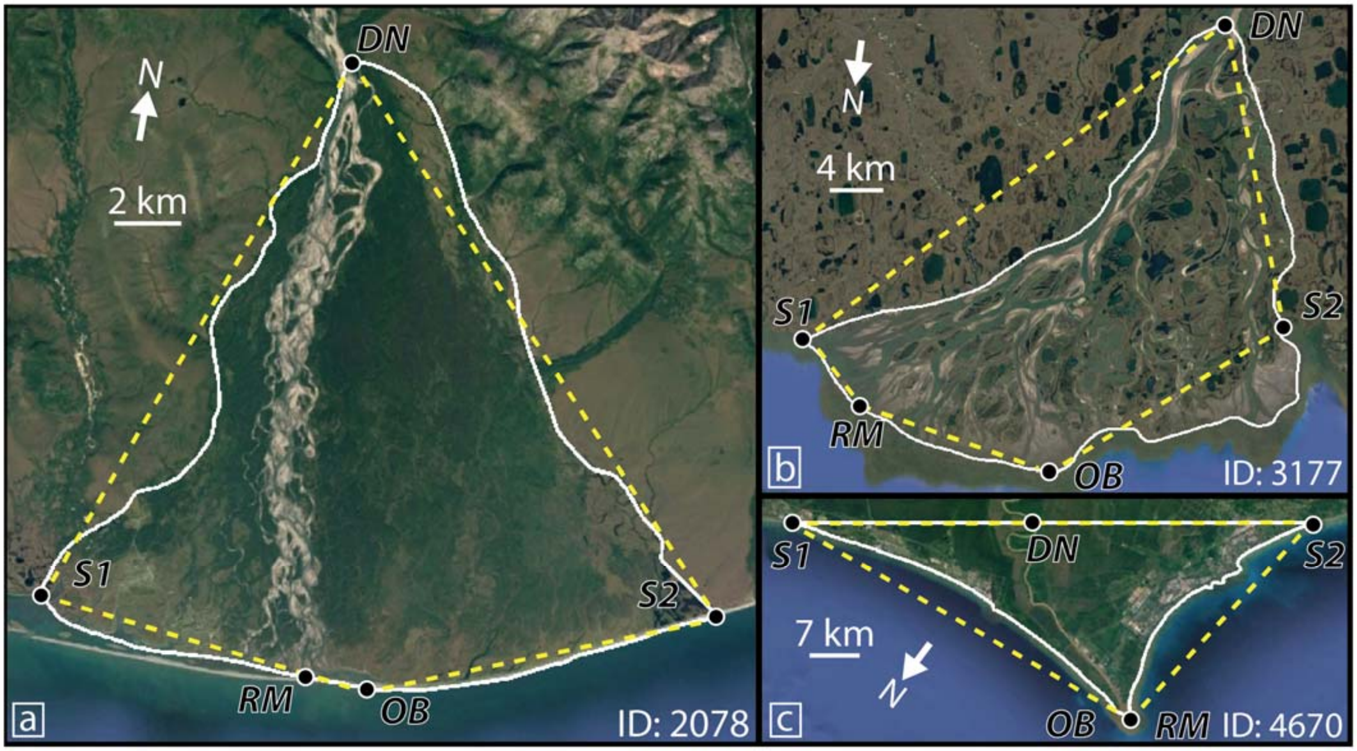

Supplementary Figure 3: Examples of delta polygons. Dashed yellow lines show the delta polygons used in this study, and white traced line is the boundary of the delta estimated by the contact between putative delta sediment and non-deltaic. (a,b,c) All three deltas show that the yellow polygon captures the first order shape of the delta.

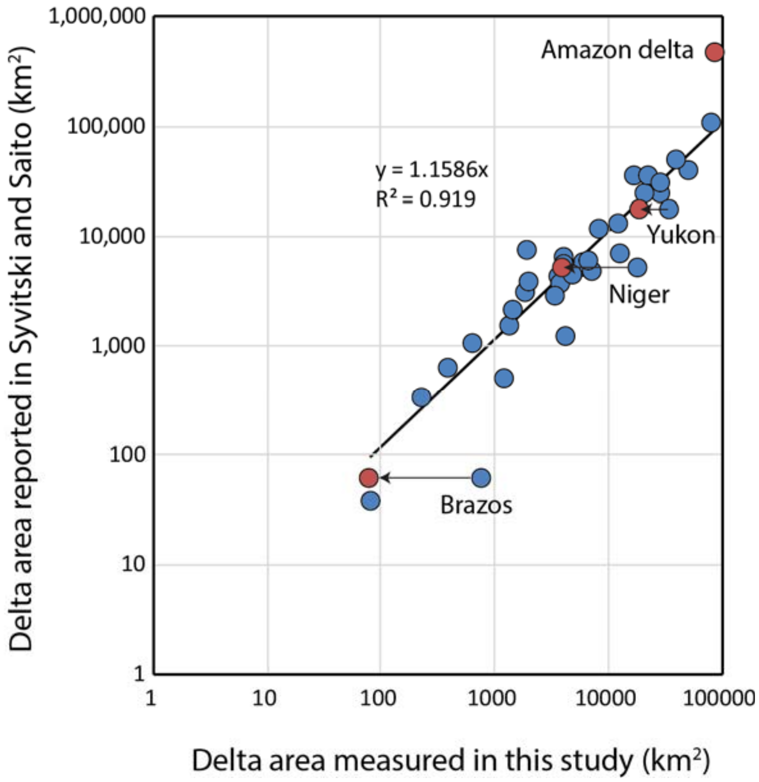

Supplementary Figure 4: Comparison of geomorphic delta area measured in this study against Syvitski and Saito. Best fit line is shown using the revised areas (shown in red) of Brazos, Niger, and Yukon deltas (see methods for details). If the original geomorphic areas are used the relationship becomes $y=1.095x$, $R^2=0.87$. In both best fit calculations, we do not include the Amazon delta since the area reported in Syvitski and Saito is one-half an order of magnitude different. Syvitski and Saito data come from reference 42 in main article.

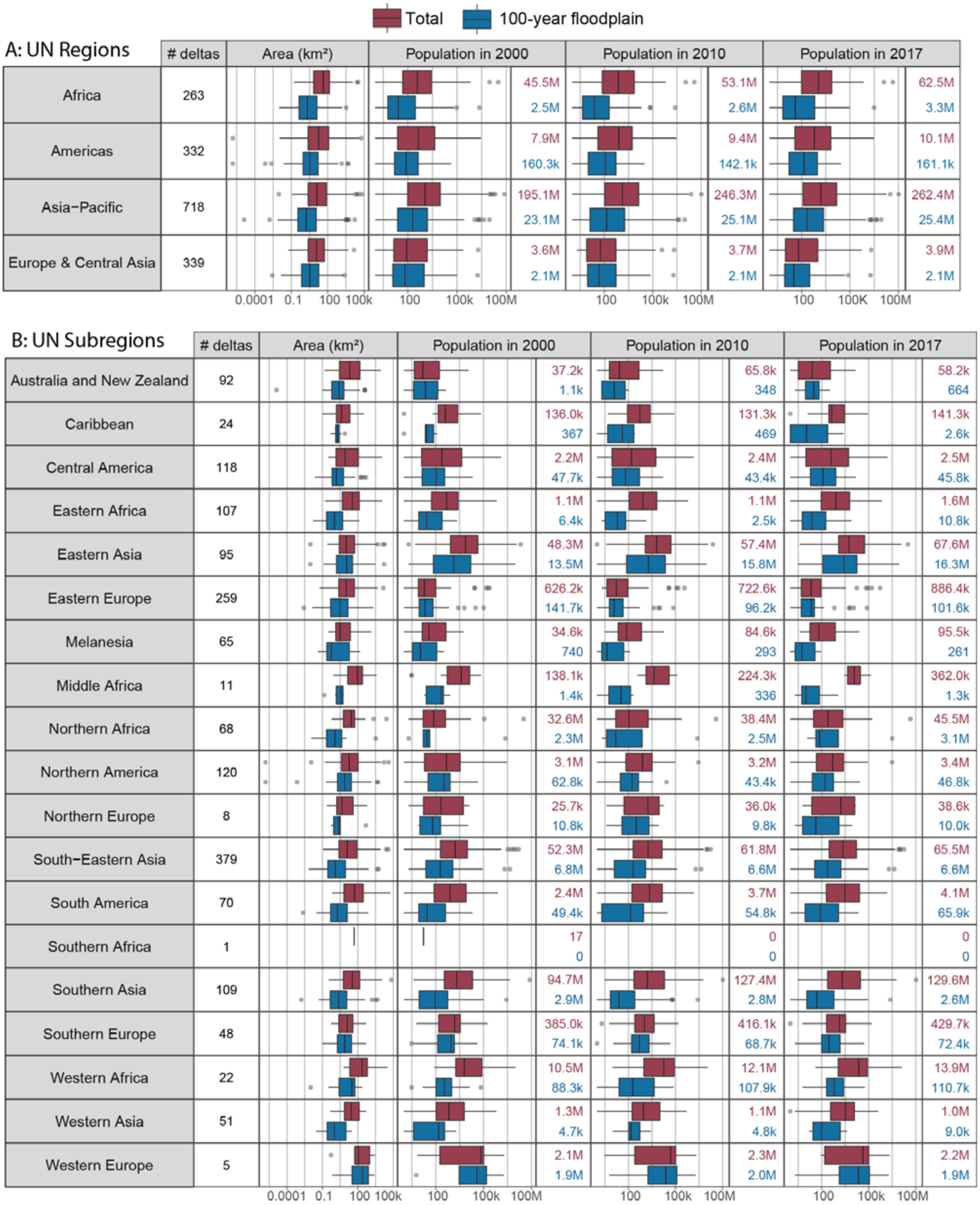

Supplementary Figure 5: Deltaic area and population broken up by United Nations regions and subregions. The whisker lengths represent the lower and upper 25% quartile distribution of all deltas within a category, and gray dots are outliers. Colored numbers refer to the total in each category

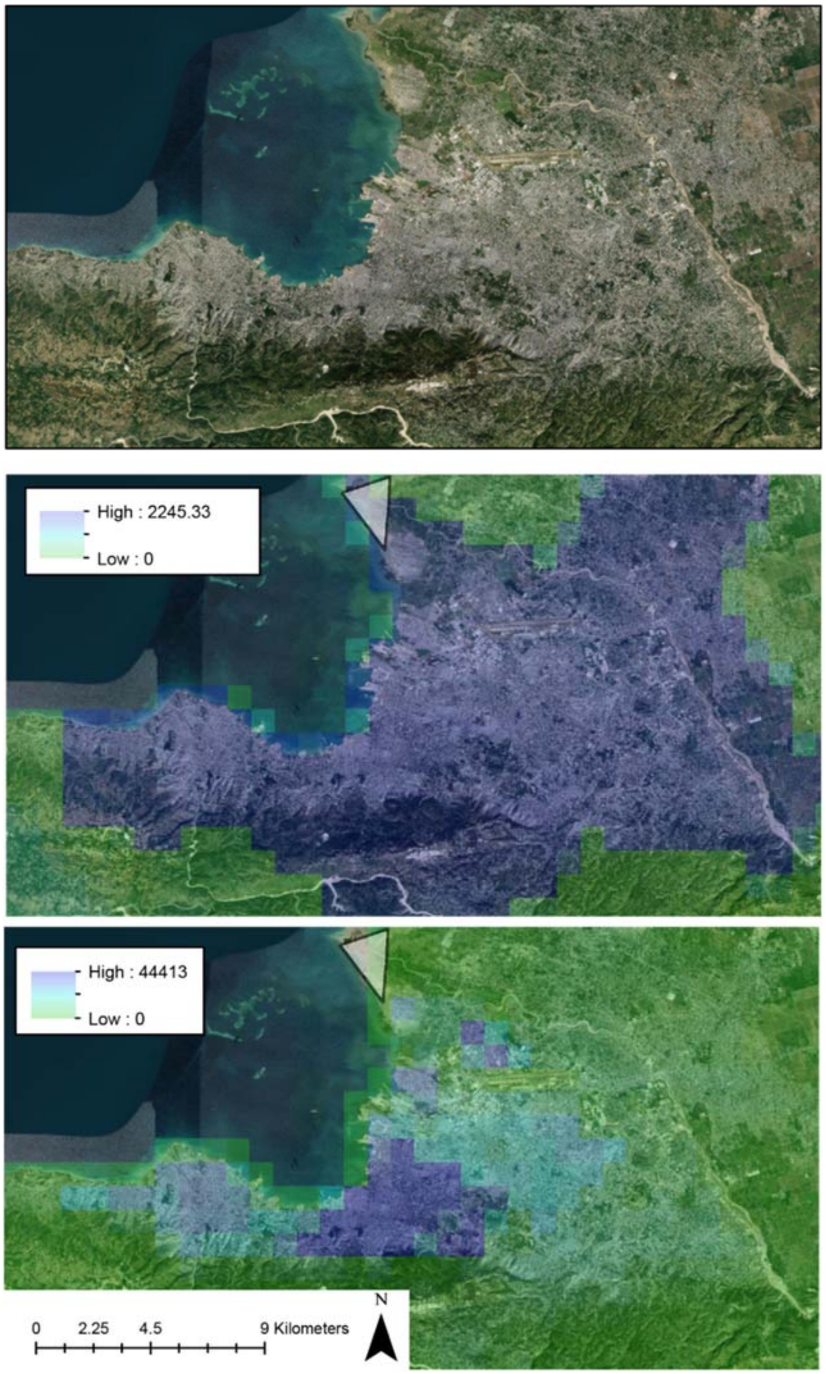

Supplementary Figure 6: Difference between LandScan and GPWv4 population datasets. Top image shows Google Earth image of Port-au-Prince, Haiti. Middle image shows the deltaic polygon at the mouth of the Grise River (ID: 1241) with the GPWv4 population raster overlain on top. Even though no people live on the delta and there is no infrastructure, GPWv4 assigns population to that location because it falls within the administrative boundary of Port-au-Prince. On the other hand, LandScan population raster, shown in the bottom image, correctly shows no people living on the delta. This is because LandScan disaggregates the population within administrative centers and uses a dasymetric, multivariable model to distribute the population.
